# Husband’s willingness-to-pay for HIV and syphilis screening at antenatal care clinic under the Thai universal coverage scheme

**DOI:** 10.1186/s12889-020-08613-9

**Published:** 2020-04-10

**Authors:** Orawan Anunsittichai, Krit Pongpirul, Thanyawee Puthanakit, Koranit Roowicha, Jirarat Kaewprasert, Wipaporn Natalie Songtaweesin, Surasith Chaithongwongwattana

**Affiliations:** 1 Department of Preventive and Social Medicine, Faculty of Medicine, Chulalongkorn University, 1873 Rama IV Rd., Patumwan, Bangkok, Thailand; 2 Center of Excellence for Pediatric Infectious Diseases and Vaccines, Faculty of Medicine, Chulalongkorn University, Bangkok, Thailand; 3 Department of International Health and Department of Health, Behavior and Society, Johns Hopkins Bloomberg School of Public Health, Baltimore, MD USA; 4 Department of Pediatrics, Faculty of Medicine, Chulalongkorn University, Bangkok, Thailand; 5 Department of Obstetrics and Gynecology, Faculty of Medicine, Chulalongkorn University, Bangkok, Thailand

**Keywords:** Willingness to pay, Sexually transmitted infection, Antenatal care

## Abstract

**Background:**

Screening for sexually transmitted infection (STI) especially HIV as early detection and treatment have been financially supported under the Thai Universal Coverage (UC) scheme since 2009 (THB140 for HIV). However, the implementation has not been evidence-based, strategic risk-based, nor economically evaluated whereas husbands who accompanied the pregnant women are likely to have a lower risk than those who did not come along. This study is aimed to determine the husband’s willingness-to-pay (WTP) for his HIV and syphilis screening tests and potential factors affecting STI screenings at the antenatal care (ANC) clinic of a tertiary hospital in Thailand.

**Methods:**

A pilot open-ended interview was conducted among 50 participants to estimate the mean and standard deviation of WTP prices for HIV and syphilis screening tests. A questionnaire was developed to obtain demographics, STI knowledge and screening history, as well as two contingent valuation methods (bidding and payment scale), using the mean WTP prices identified from the pilot study as a starting WTP with ¼SD step-up/down. The survey of 200 randomly selected husbands of pregnant women was conducted at King Chulalongkorn Memorial Hospital from April to June 2018. Descriptive statistics and logistic regression were used for data analysis.

**Results:**

During the study period, 597 pregnant women received their first ANC. Of 368 accompanying husbands, 200 were enrolled in the study. Their median age was 31 (IQR 27–36) years old and 67% had a first child. Eighty-eight percent of the participants were willing to test for the STIs. Based on the bidding method, WTP prices for HIV and syphilis screening tests were US$14.5 (IQR 12.4–14.5) and US$9.7 (IQR 10–12), respectively. The payment scale method suggested approximately three-quarters of the WTP prices from the bidding method.

**Conclusions:**

The husbands who accompanied their pregnant wives to the ANC clinic showed positive behaviors according to the propitious selection theory. They tend to cooperate well with STI testing and are willing to pay at least two times the price of the STI screening tests. The financial support to promote STI screenings should be reconsidered to cover other groups with higher sexual behavior risks and less WTP.

## Background

Sexually Transmitted Infections (STIs) including HIV and syphilis are major public health burdens worldwide. The Joint United Nations Programme on HIV/AIDS (UNAIDS) reported 36.9 million people living with HIV, of which 180,000 were children aged 0 to 14 years old who were newly infected in 2017 [1]. In 2016, the prevalence of maternal syphilis was 0.69% worldwide which caused congenital syphilis rates of 473 per 100,000 live births [2]. In Thailand, the prevalence of maternal syphilis was increasing from 0.1 in 2015 to 0.2 in 2017 [3]. To eliminate mother-to-child transmission (MTCT) both of HIV and syphilis, STIs screening test at antenatal care clinic (ANC) is recommended to detected newly infection and refer to received early treatment.

The World Health Organization (WHO) has set specific goals for eliminating MTCT; HIV by 2020, syphilis by 2030 and ending AIDS as a public health threat by 2030. To achieve these goals, the Couple HIV Test and Counseling (CHCT) program should be promoted at ANC to find incidental infections and promote early treatment. UNAIDS estimated that US$26.2 billion will be required for the global HIV response in 2020 [4] whereas funding for the HIV response in the low- and middle-income countries has come from domestic rather than international sources. This is because high-income countries have reduced funding for the HIV response with a 7% decrease reported between 2015 and 2016 [4]. Because funding for HIV tends to be decreasing, the willingness-to-pay (WTP) for STI screening tests should be explored as evidence for policymaker if out of pocket planning is needed.

Among late-presenting pregnant women to ANC, incidental HIV infections during pregnancy and poor antiretroviral therapy (ART) adherence have been reported as high-risk for MTCT in Thailand [5]. By providing treatment using three-drug regimens and HIV-1 integrase inhibitors among incidental HIV infections during pregnancy which have a high maternal viral load, HIV transmission rates can be reduced from 9.0 to 3.5% [6]. As a result of many interventions to eliminate MTCT in Thailand, the prevalence of HIV among pregnant women decreased from 2% in the 1990s to 0.6% in 2015. In 2016, WHO announced Thailand to be the first country in Asia-Pacific to achieve a decrease of MTCT to below 2% [7].

In Thailand, although more than 98% of pregnant women received STI screening tests during ANC services [8], the CHCT program piloted in 2009 achieve uptake of only 39% [9]. Since then, the National Health Security Office (NHSO) has supported the CHTC program by covering expenses for Thai pregnant women and their husbands to receive HIV screening tests during ANC services [10]. This policy has not been evidence-based, strategic risk-based, or passed any economic evaluation, particularly among husbands accompanying their pregnant wives. According to propitious selection theory, people who have positive health behaviors such as smoking avoidance, seat-belt use while driving, and receiving annual health check-ups are more likely to obtain health insurance and tend to have higher WTP for their healthcare services than those who do not [11]. Likewise, husbands accompanying their pregnant wives are therefore likely to have lower risk and are more willing to pay for their health care services. To increase the uptake of CHTC while financially sustaining the MTCT elimination programs, policymakers should consider economic evaluation evidence and potential budget impact for the most efficient use of the limited resource.

For the economic evaluation, WTP has been adopted by health economists who were concerned with identifying prospective public valuations of health care interventions, and the applications of the technique in this context have proliferated [12]. WTP for STI screenings has been explored among various population groups but not among husbands who accompanied the pregnant women (Table 1). Results showed that the subjects were willing to pay for their STI screening tests.

**Table 1:** Literature on Willingness-to-Pay for HIV Screening Test

Study	Population	Study Design	Country	WTP Method	Local Price	WTP	GDP 2016
Huanmiao Xun (2013) [13]	1151 of MSM*, female sex workers and VCT* clients	Cross-sectional survey	China	Not specified	US$17	Median US$4.8 to US$8.1	11,199.1
Xu Y (2013) [14]	MSM at a VCT site at the Beijing Jingcheng Skin Disease Hospital	Cross-sectional survey	China	Open-ended	US$16	Median US$8	11,199.1
Li J (2015) [23]	511 of people seeking counsel and HIV* test, STD* clinic patients, university students, migrant people, female sex workers, MSM and injecting drug users	Cross-sectional survey	China	Not specified	Unknown	84.1% were willing to pay for HIV antibody saliva rapid test	11,199.1
Long Hoang Nguyen (2016) [15]	365 VCT clients in Ha Noi and Nam Dinh province	Cross-sectional survey	Vietnam	Open-ended for pilot study then Bidding	US$20	Mean US$7.75 (2013)	202.6
Bach Xuan Tran (2016) [24]	1016 MMT* patients in Hanoi and Nam Dinh	Cross-sectional survey	Vietnam	Bidding and Open-ended	US$20	Mean US$17.9	202.6
Forsythe S (2002) [16]	2 health care centers	Cross-sectional survey	Kenya	Payment Scale and Bidding	US$2–6	At least US$2	70.5
Uzochukwu B (2011) [17]	250 of undergraduate students of two tertiary institutions	Cross-sectional survey	Nigeria	Bidding and Open-ended	Free of charge	Mean US$3.2	405.1
Maria Jose Bustamante (2017) [18]	147 MSM and 45 transgender women	Cross-sectional survey	Peru	Not specified	US$10	Mean US$5	192.1

* *VCT* Voluntary Counseling and Testing; *MSM* Men who have sex with men; *STD* Sexually transmitted disease; *HIV* Human immunodeficiency virus; *MMT* Methadone Maintenance Treatment

As evidence of WTP for STI screening tests among husbands of pregnant women in the setting of ANC service has been limited, this study was aimed to determine (1) the husband’s WTP for STI screening tests and (2) potential factors affecting STI screenings among accompanying husbands at ANC clinic of a large tertiary hospital in Bangkok, Thailand.

## Methods

This study was conducted at an ANC clinic of King Chulalongkorn Memorial Hospital, Bangkok, Thailand. It was approved by the Institutional Review Board of the Faculty of Medicine, Chulalongkorn University, Bangkok, Thailand (IRB No.028/61) and registered on the Thai Clinical Trials Registry (TCTR20180307003).

### Estimating initial WTP prices

In March 2018, 50 conveniently sampled husbands of pregnant women were asked how much they were willing to pay for HIV and syphilis screening tests. They were not aware that the hospital prices of HIV and syphilis tests were US$4.5 or 140 Thai Baht (US$1 = 31 Thai Baht) and US$3.2 or 100 Thai Baht, respectively. The survey revealed the mean starting WTP prices of US$14.5 (SD 281.7) for HIV and US$9.7 (SD 270.8) for syphilis screening tests. These mean prices were chosen as the initial WTP prices with stepping up or down by ¼SD (US$2.2).

### Questionnaire development

The questionnaire contained three sections. The first section obtained demographic information including age, educational level, and history of having a child. The second section obtained the history of HIV and/or syphilis screening tests and knowledge of HIV prevention using the United Nations General Assembly Special Session on HIV/AIDS (UNGASS) score (0–3, low; 3–4, moderate; 5, high). Based on the evidence from literature, the age [16], income [13, 16], educational level [16, 18–20], history of having a child [16], history of STI screen tests [16, 18, 21], and knowledge of HIV prevention [16] were considered potential factors affecting WTP and willingness to test for STIs. Other potential determinants such as the number of ANC visits [20] were excluded because they were beyond the scope of this survey.

**Fig. 1:**
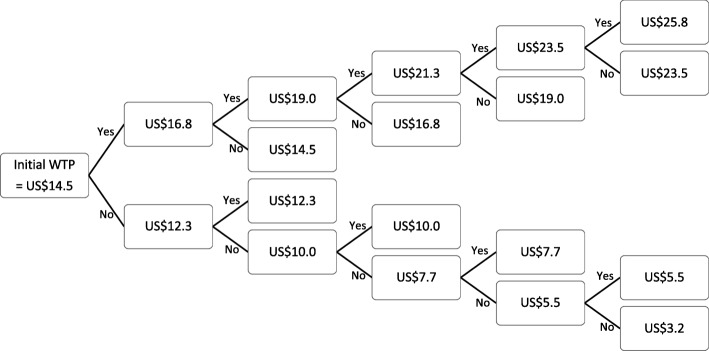
Willingness-to-Pay for HIV Screening Test using the Bidding Method

**Fig. 2:**
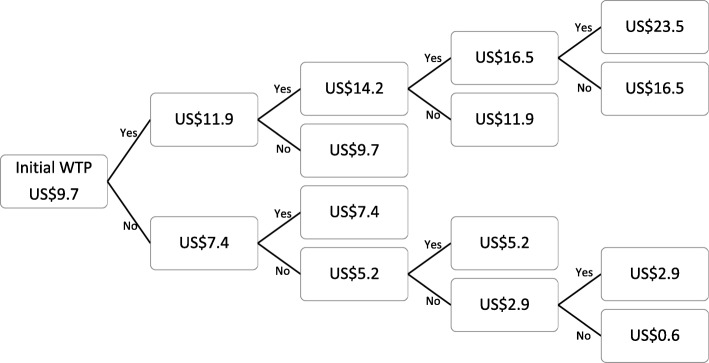
Willingness-to-Pay for Syphilis Screening Test using the Bidding Method

The third section asked about the willingness to test and the WTP for HIV and syphilis screening tests, using two contingency valuation methods: payment scale and bidding. For the payment scale method, the range of WTP price for HIV screening test was US$3.2 to US$25.8 (100 to 800 Thai Baht) whereas that of syphilis was US$2.5 to US$18.7 (77.5 to 579.7 Thai Baht). The husbands were informed of all available WTP options before choosing the price that they were willing to pay for the tests. For the bidding method, the participants were asked a series of Yes/No questions about their WTP specific prices for HIV and syphilis tests. First, they were asked whether they were willing to pay US$14.5 (450 Thai Baht) for HIV and US$9.7 (300 Thai Baht) for syphilis screening tests. Depending on their responses, the interviewer presented the US$2.2 (70 Thai Baht) higher bid for respondents answering “Yes” and the US$2.2 (70 Thai Baht) lower bid for respondents answering “No”. The question was repeated for the maximum of five and four rounds for HIV and syphilis tests, respectively (Fig. 1 and Fig. 2).

### Data collection

At the first ANC visit, all husbands accompanying their pregnant wives were invited to participate in the study and informed about the ANC service process. After obtaining the written informed consent from the participants, they were allocated into two groups of WTP methods—payment scale or bidding—randomly assigned by date (payment scale on odds day and bidding on even day). We used face-to-face interviews to obtain information in the questionnaire as described above. Then, the husbands received blood drawn for HIV and syphilis screening tests; free of charge for HIV and US$3.2 (100 Thai Baht) for an optional syphilis screening test.

### Sample size estimation

The sample size of this study was calculated by using the finite population mean formula [14]. We estimated the sample size by using the value gained from the pilot study in March 2018, population size (N) was 188 (number of accompanying husband), standard deviation (σ) 281.7 (Thai Baht), error (d) 0.1 and alpha (α) 0.5; at least 188 subjects were anticipated. To elicit WTP for STI screening tests using the two methods, 200 subjects were enrolled in the study by convenience sampling and randomly separated into two equal method groups.

### Statistical analysis

Interval variables including the WTP prices were analyzed by using the median (interquartile range, IQR) whereas categorical variables including the number of husbands who were willing to receive STI screening tests were analyzed by using percentages. The odds ratio was used to assess the association between an independent categorical variable and the willingness to get STI tests. Multivariate logistic regression analysis was used to determine factors associated with willingness to test for the STIs. SPSS version 22 was used to analyze the data and statistical significance was set at *P* < 0.05.

## Results

From April to June 2018, 597 pregnant women came to the first ANC care service, 368 husbands (62%) accompanied them. Of these, 200 husbands were enrolled in the study. The demographics data of the participants were shown in Table 2. The median (IQR) age was 31 (27–36) years old, 67% of the husbands reported that this ANC visit was for their wife’s first pregnancy, 44% had been tested for STIs and 40% of husbands had university-level education.

**Table 2:** Characteristics of Participating Husbands

Characteristics	Total (*n* = 200)
Age (Median, IQR)	31 (27–36)
Income (Median, IQR)	581 (484–968)
History of having a child (n, %)	66 (67.0)
History of HIV and/or Syphilis Screening Tests** (n, %)	88 (44.0)
Educational levels (n, %)	
≤ Junior high school	36 (18.0)
High school/Diploma	84 (42.0)
University	80 (40.0)
Knowledge about HIV Prevention** (n, %)	
Low (Score 0–3)	8 (4.0)
Moderate (Score 3–4)	81 (40.5)
High (Score 5)	111 (55.5)

*US$1 = 31 Thai Baht

### WTP for HIV and syphilis Testings

Based on the bidding method, the husbands were willing to pay US$14.5 (12.3–14.5) for HIV and US$9.7 (9.7–11.4) for syphilis screening tests, respectively. The payment scale method suggested lower WTP for both: US$10 (5.5–14.5) and US$7.4 (5.2–9.7), respectively (Table 3). Husbands who had never had a previous child compared to husbands who had at least one child were willing to pay more for both HIV (95% CI; 20.14, 107.93) and syphilis (95% CI; 14.57, 86.45) screening tests. Husbands with a university-level education were more willing than those with high school/diplomas to pay more for both HIV (95% CI; 10.22, 94.95) and syphilis (95% CI; 17.05, 85.87).

**Table 3:** Willingness-to-Pay for HIV and Syphilis Screening Tests

	All Husbands (*n* = 200)	Median (IQR) (US$)	Not willing to test (*n* = 24)
		Willing to test (*n* = 176)	
HIV Screening			
- Bidding	14.5 (12.2–14.5)	14.5 (12.3–14.5)	14.5 (11.7–14.5)
- Payment Scale	9.7 (5.5–14.5)	10.0 (5.5–14.5)	12.3 (8.9–15.1)
Syphilis Screening			
- Bidding	10 (9.7–11.4)	9.7 (9.7–370)	9.7 (9.1–10.8)
- Payment Scale	7.4 (5.2–9.7)	7.4 (5.2–9.7)	9.7 (6.3–14.2)

*US$1 = 31 Thai Baht

### Willingness to test for STIs

Among the husbands who accompanied the pregnant women, 88.0% were willing to test for STIs. Of 24 unwilling husbands, 17 husbands were already tested recently, four husbands preferred to test at other health care services, two husbands thought they had no risk, and one husband was fearful of a needle. Husbands who had been tested for STIs were significantly more willing to test than those had never had (odds ratio 4.82; 95% CI; 1.77, 13.08) (Table 4). Husbands who had had at least one child were willing to test more than those who had never had a child (odds ratio 0.81; 95% CI; 0.30, 2.14). Husbands with a university-level education were less willing to have STI screening tests than those with lower educational levels (odds ratio 0.76; 95% CI; 0.32, 1.80). Husbands with higher knowledge scores were more willing to have STI screening tests than those with less than high knowledge scores (odds ratio 1.55; 95% CI; 0.66, 3.66).

**Table 4:** Factors Associated with Willingness to Test for HIV and Syphilis

Factors	Adjusted Odds Ratio	95% Confidence Interval
Age	0.93	(0.86, 1.01)
Educational levels: University	0.76	(0.32, 1.80)
History of having a child: Never	0.81	(0.30, 2.14)
History of HIV and/or Syphilis Screening Tests	4.82	(1.77, 13.08)
Knowledge about HIV Prevention: High Score	1.55	(0.66, 3.66)

## Discussion

To our knowledge, this is the first study to determine WTP for STI screening tests among husbands accompanying their pregnant wives at ANC clinic whereas other studies have explored this among men who have sex with men, commercial sex workers, as well as voluntary counseling and testing clients as shown in Table 1. Three contingent valuation methods have commonly been used to find the WTP price for STI screening tests. The payment scale method suggested lower WTP prices than the bidding method whereas the open-ended and bidding methods suggested similar WTP prices for the STI screening tests. Husbands who accompanied their pregnant wives at the ANC clinic of our institution showed the WTP at least double the hospital price for the STI screening tests.

In our study, based on the bidding method, the husbands were willing to pay US$14.5 and US$9.7 for HIV and syphilis screening tests, respectively while the payment scale method suggested lower WTP for both (US$10 and US$7.4). However, previous studies showed lower WTP prices among high-risk populations for an HIV test: US$5 in Peru [18], US$7.75 in Vietnam [15], US$2 in Kenya [16] and US$4.8–8.1 in China [13]. The general population in China was willing to pay US$8 [17] and students in Kenya US$3.2 [19]. The WTP prices for an HIV screening test varied across studies may be because of different HIV prevalence, health literacy level, and socio-economic factors.

While previous studies explored the WTP value for only one disease, this study explored the WTP prices for HIV and syphilis screening tests simultaneously. As the HIV/AIDS is perceived by a layperson as relatively more severe than syphilis so the WTP amount should be reflective, this study showed the higher WTP price of HIV than that of syphilis screening tests, suggesting good concurrent validity of the findings.

In Thailand, the HIV screening test has been financially supported by the Thai Universal Coverage Scheme since 2009; the pregnant women and their husbands who attend ANC service at any public health care facility are eligible for an HIV screening test free of charge two times per year. Husbands who accompanied their wives to attend ANC service are not only considered ‘family men’ but also financially viable and have time to spend with the loved ones, suggesting a lower risk of STI than those at lower socioeconomic status. Therefore, government subsidization of the laboratory expenses should be for the poor and/or high-risk.

In this study, 88% of the accompanying husbands were willing to test for STIs. This is similar to other studies. Batte et al. conducted a survey in Uganda and reported 98.9% of pregnant partners to receive CHTC whereas only 42.4% were tested when coming separately [20]. Moreover, Thirumurthy et al. in Kenya attributed HIV self-testing of partners through pregnant women; the result showed 91% of the pregnant women gave the self-tests to their partner but only 51% were tested [22], suggesting that husbands tended to receive HIV testing when they were approached as a couple.

History of STI test was significantly associated with willingness to test; Xu et al. reported the association between history of STI testing, education, risk behavior and willingness to test among men who have sex with men and female sex workers [18]. The associated factors may be different across population groups. Batte et al. in Uganda reported the significant association between the number of ANC services and willingness to test [20] but this study surveyed only the first ANC visit so the impact of ANC visits could not be investigated.

Barriers to STI screening at our ANC clinic were similar to previous studies. Musheke et al., for example, reported self-perception of no risk, fear of secret being revealed, stigma, cost of STIs testing and gender equality [21]. In Thailand, Lolekha et al. conducted a survey in the pilot CHTC program and revealed that the husbands of pregnant women who were unwilling to test for STI reported self-perception of no risk, intention to be tested at another facility, fear of the needles, and already know their HIV status to be barriers to STI screening uptake [9]. Our study showed 44% of husbands who received STI testing previously and 88% were willing to test with their pregnant wives. According to the propitious selection theory, supported by our findings, husbands who accompanied their wives to ANC services were more likely to cooperate with the CHTC program.

Some limitations should be noted. This study was conducted at an ANC clinic of a tertiary care hospital and all data were collected from randomly selected husbands of pregnant women receiving their first ANC service so the findings might not be generalizable to other settings. Husbands who participated in the study may be more willing to cooperate and able to pay for their STI screening tests than those in lower socioeconomic contexts. We did not have information about one-third of husbands who did not come to the ANC clinic and might be of lower socioeconomic status and/or less health concerned. The study was conducted at a university hospital, thus not representative of Thailand, as Bangkok populations, in general, have higher incomes than those in rural areas. Additionally, individuals who come to university hospitals usually agree to pay medical care costs out-of-pocket as opposed to patients who go to the Ministry of Public Health hospitals under the Universal Coverage Scheme. For further studies, the survey should be conducted at multiple ANC service settings nationwide. To represent the general population, the survey should be done in husbands attending and not attending ANC services with their pregnant wives. Also, the survey should cover all population groups to provide better evidence policymakers to allocate more resources to people who have higher risk but lower ability to pay. For the reliability of the willingness to pay, comparing two diseases and two contingent valuation methods are recommended.

## Conclusion

The husbands who accompanied their pregnant wives to the ANC clinic showed positive behaviors according to the propitious selection theory. They tend to cooperate well with STI testing and are willing to pay at least two times the price of the STI screening tests. The financial support to promote STI screenings should be reconsidered to cover other groups with higher sexual behavior risks and less WTP.

## Data Availability

The datasets used and/or analyzed during the current study are available from the corresponding author on reasonable request.

## Abbreviations

AIDS: Acquired immunodeficiency syndrome
ANC: Antenatal clinic
ART: Antiretroviral therapy
CHTC: Couple HIV test and counseling
HIV: Human immunodeficiency virus
IQR: Interquartile range
IRG: Institutional review board
MTCT: Mother-to-child transmission
NHSO: National health security office
SD: Standard deviation
STI: Sexually transmitted disease
TCTR: Thai clinical trial registry
UC: Universal coverage
UNAIDS: the joint united nations programme on HIV/AIDS
UNGASS: United nations general assembly special session on HIV/AIDS
WHO: World health organization
WTP: Willingness to pay
